# Synthesis of Pyrazole-Thiobarbituric Acid Derivatives: Antimicrobial Activity and Docking Studies

**DOI:** 10.3390/molecules21101337

**Published:** 2016-10-09

**Authors:** Yaseen A. M. M. Elshaier, Assem Barakat, Bander M. Al-Qahtany, Abdullah Mohammed Al-Majid, Mohamed H. Al-Agamy

**Affiliations:** 1 Pharmaceutical Organic Chemistry Department, Faculty of Pharmacy, Al-Azhar University, Assuit 71524, Egypt; yaseenorganic@yahoo.com; 2 Department of Chemistry, College of Science, King Saud University, P. O. Box 2455, Riyadh-11451, Saudi Arabia; b.m.s.m@hotmail.com (B.M.A.-Q.); amajid@ksu.edu.sa (A.M.A.-M.); 3 Department of Chemistry, Faculty of Science, Alexandria University, P. O. Box 426, Ibrahimia, Alexandria 21321, Egypt; 4 Microbiology and Immunology Department, Faculty of Pharmacy, Al-Azhar University, Cairo 11884, Egypt; malagamy@ksu.edu.sa; 5 Division of Microbiology, Pharmaceutics Department, College of Pharmacy, King Saud University, P.O. Box 2457, Riyadh 11451, Saudi Arabia

**Keywords:** pyrazole, thiobarbituric acid, antimicrobial activity

## Abstract

A one-pot reaction was described that results in various pyrazole-thiobarbituric acid derivatives as new pharmacophore agents. These new heterocycles were synthesized in high yields with a broad substrate scope under mild reaction conditions in water mediated by NHEt_2_. The molecular structures of the synthesized compounds were assigned based on different spectroscopic techniques. The new compounds were evaluated for their antibacterial and antifungal activity. Compounds **4h** and **4l** were the most active compounds against *C. albicans* with MIC = 4 µg/L. Compound **4c** exhibited the best activity against *S. aureus* and *E. faecalis* with MIC = 16 µg/L. However, compounds **4l** and **4o** were the most active against *B. subtilis* with MIC = 16 µg/L. Molecular docking studies for the final compounds and standard drugs were performed using the OpenEye program.

## 1. Introduction

**Figure 1:**
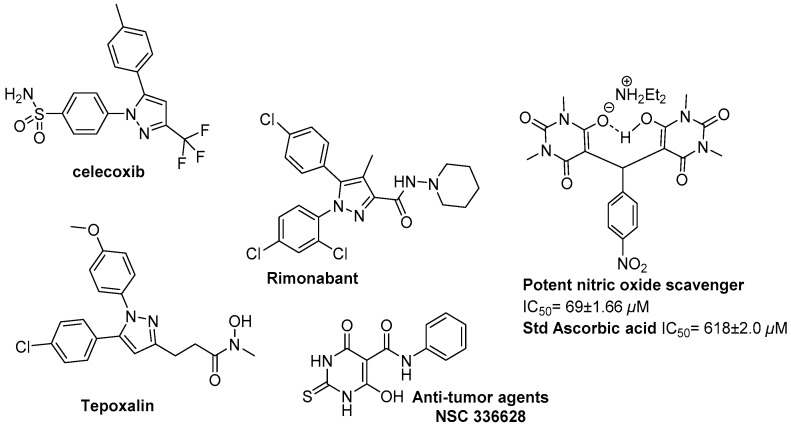
Biologically active pyrazole and barbituric acid scaffolds.

Pyrazole heterocycles are a core structure for pharmaceutical targets in the synthetic and natural products [1,2,3]. For example, Celecoxib, SC-558, and tepoxalin were reported as cyclooxygenase 2 inhibitors [4,5], and rimonabant was identified as reducing obesity for example cannabinoid-1 inverse agonists (Figure 1) [6,7]. Additionally, pyrazole derivatives display usefulness in the fields of luminophores, fluorescence applications, agricultural research, and drug discovery [8,9,10,11,12,13,14,15]. Pyrazoles fused with other privileged structures may possess some promising pharmacological and other activities. Pyrazole and pyrazole-based derivatives have been reported as potential CB2 receptor ligands with antagonist/inverse agonist properties [16], dual inhibition of CCR5/CXCR4 HIV entry [17], and reverse transcriptase HIV-1 non-nucleoside reverse transcriptase inhibitors [18]. Pyrimidine-2,4,6-trione derivatives are an important class of nitrogen heterocycles that have attracted more attention in the last decade: due to their use as a precursor for the construction of condensed heterocyclic systems, they represent an interesting pharmacophore for pharmaceutical products [19,20,21,22,23,24,25,26,27,28,29,30,31,32]. They have utility as anticancer agents, enzyme inhibitors, neuromodulators, antibiotics, herbicides, and plant growth regulators, antibacterial, and anti-oxidant agents (Figure 1). Structure activity relationship SAR development led to the discovery of new, selective, and potent inhibitors that have attracted much interest from chemists. Molecular docking plays an important role in the rational design of drugs. In the field of molecular modeling, docking is a tool that predicts the best orientation of one molecule to a second when bound to each other to form a stable complex. Molecular docking can be defined as an optimization problem that would describe the “best-fit” orientation of a ligand that binds to a particular protein of interest to allow it to perform reliable virtual screening processes, and help us to understand the mechanism of action for tested compounds [33,34].

In this paper, we synthesized a new series of pyrazole-thiopyrimidine–rione derivatives via *a* one-pot multi-component reaction in aqueous media to identify new drugs as antimicrobial agents. We subjected our target compounds to molecular docking study with different target proteins to explore their mode of action.

## 2. Results

**Scheme 1:**
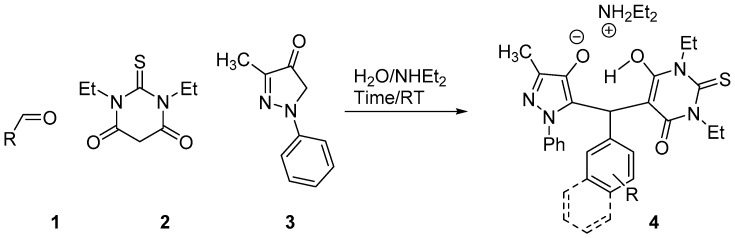
Substrate scope of the cascade reaction: variation of pyrazole-thiobarbituric acid adducts.

As part of our continuing efforts on the synthesis of bioactive scaffolds using green protocols, we envisioned that pyrazole-thiobarbituric acid derivatives having various substituents were achieved in high yields (63%–88%), as shown in Scheme 1, Table 1. Cascade Aldol-Michael addition of *N*,*N*-diethyl thiobarbituric acid, 3-methyl-1-phenyl-1*H*-pyrazol-5(4*H*)-one and aldehyde was mediated by aqueous NHEt_2_ to afford **4a**–**o**. Notably, the present one-pot transformation provides an efficient method for the flexible and rapid synthesis with substrate tolerance of pyrazole-thiobarbituric acid derivatives. The chemical structures of all the synthesized compounds were assigned with the aid of physical and spectroscopic methods.

**Scheme 2:**
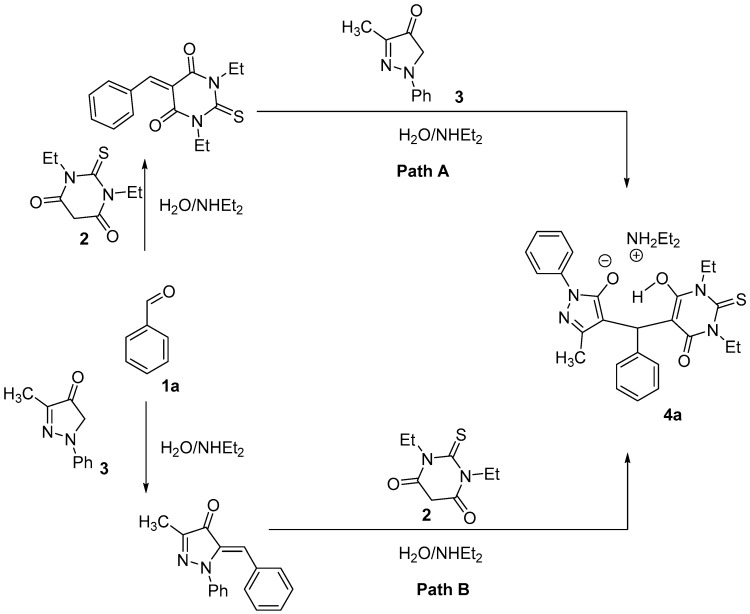
Possible mechanism of the tandem Aldol-Michael reaction.

A tandem Aldol-Michael reaction is shown in Scheme 2. In the first step of the reaction, olefin is produced by Aldol condensation between aryl aldehyde **1** and **2** to give an intermediate that acts as a Michael acceptor and is attached by **3** (the Michael donor) to afford the final product **4a** (Bath A). Alternatively, Aldol condensation between aryl aldehyde **1** and **3** gives an intermediate that acts as a Michael acceptor and is attached by **2** (the Michael donor) to afford the final product **4a** (Bath B). [19].

## 3. Discussion

### 3.1. Antimicrobial Activity

Results of the biological activity were displayed in Table 2; results are expressed as µg/L inhibition.

#### 3.1.1. Antibacterial Activity against Gram-Positive Bacteria

The antibacterial activity of synthesized compounds was elucidated against six strains including *E. faecalis* ATCC29212*, S. aureus* ATCC 29213, *E. coli* ATCC 25922, *B. subtilis* ATCC 10400, *P. aeruginosa* ATCC 27857, and *P. s vulagris* ATCC 6380. Their activities were compared with the known Ciprofloxacin.

The results summarized in Table 2 show that all compounds are sensitive to the tested strains including *E. faecalis*, *S. aureus*, and *B. subtilis* except for compounds **4d**–**f** and **4o**, which were not active against *S. aureus* and *E. faecalis*, respectively. Compound **4c** was the most active compound against *S. aureus* with an MIC value of 16 µg/L. In addition, it exhibited good activity against *E. faecalis* and *B. subtilis*, with MIC values of 16 µg/L and 32 µg/L, respectively*.* Compound **4j** showed activity against *E. faecalis* (similar to **4c**) with MIC values of 16 µg/L. Compounds **4l** and **4o** showed activity against *B. subtilis* with MIC values of 16 µg/L. Compounds **4a**, **4b**, **4i**, **4m**, and **4n** showed moderate activity against all bacteria with MIC values of 32 µg/L. Compound **4h** exhibited moderate activity against *E. faecalis* and *S. aureus* with MIC values of 32 µg/L and weak activity against *B. subtilis* with MIC values of 64 µg/L. Compound **4k** exhibited moderate activity against *E. faecalis* and *B. subtilis* with MIC values of 32 µg/L and weak activity against *S. aureus* with MIC values of 64 µg/L. Compounds **4d**, **4e**, and **4f** were the least active compounds against the tested bacteria. The synthesized compounds showed no activity against *P. aeruginosa*, *E. coli*, or *P. vulgaris*.

#### 3.1.2. Antifungal Activity

The new pyrazole-thiobarbituric acid derivatives were evaluated for their antifungal activity against fungi) *C. albicans* ATCC 2091) by the diffusion method and serial dilution method (BSAC, 2015). Their activities were compared with the known antifungal agent Fluconazole. Compounds **4h** and **4l** were the most active compounds against *C. albicans*, with MIC values of 4 µg/L, followed by compounds **4a** and **4o** with MIC values of 8 µg/L. The order of activity was compounds **4b**, **4c**, **4k**, **4m**, **4n**, and **4j** with MIC values of 16 µg/L, then compounds **4f**, **4g**, and **4i** with MIC values of 32 µg/L. Compounds **4d** and **4e** were the least active compounds with MIC values of 36 µg/L. Among those compounds most active as antimicrobial, we can conclude that the aryl part is either incorporated by the electron withdrawing group (EWG) at the para position or is replaced by heterocyles (compound **4o**).

#### 3.1.3. Molecular Docking as Antifungal

Comparative consensus score of synthesized compounds were listed in Table 3. The synthesized compounds and fluconazole were docked with Lanosterol 14 α-demethylase (CYP51A1) (PDB ID 4WMZ) [35].

**Figure 2:**
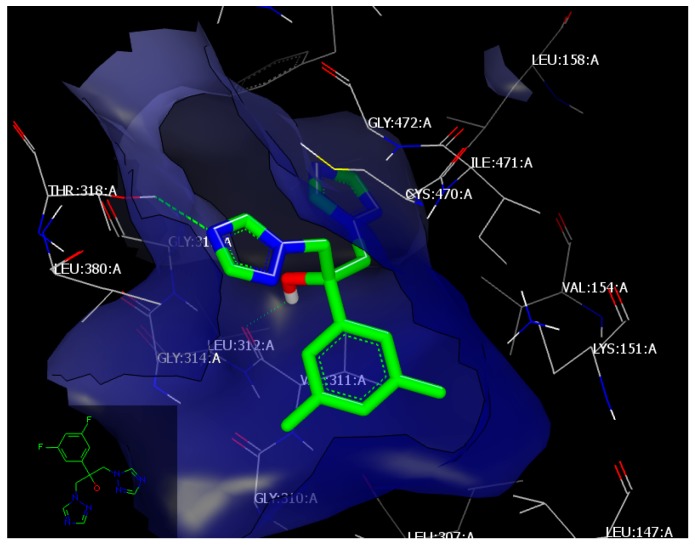
Visual representation of fluconzol docked with 4WMZ, showing two hydrogen bonding interactions with THR 318:A and LEU 312:A, as shown by Vida.

**Figure 3:**
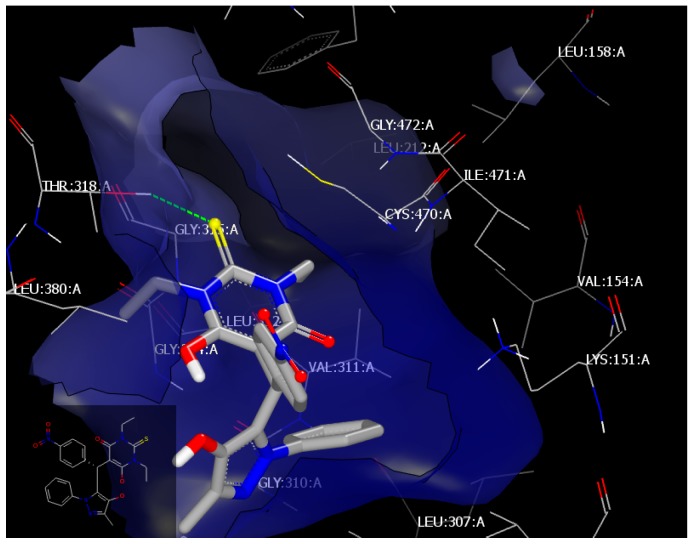
Visual representation of **4h** docked with 4WMZ, showing hydrogen bonding interactions with THR 318:A, as shown by Vida.

The standard drug, fluconazole, with a consensus score of 57, forms two hydrogen bonds with the receptor; with THR318:A through the nitrogen of triazole moiety (HB acceptor) and with Val 311:A through the hydroxyl functionality (HB donor) (Figure 2). Compound **4l**, with a consensus score of 48, interacts with the same receptor through hydrophobic–hydrophobic interaction without HB formation. Compound **4h**, with a consensus score of 55, forms a hydrogen bond with receptor THR 318:A (as fluconazole did) through the sulfur of the pyrimidin moiety (HB acceptor), (Figure 3).

Our hypothesis was directed at the study of another kind of protein as these compounds may have a mode of action that differs from the fluconazole mechanism. After intensive study, synthesized compounds presented docking interaction with dihydrofolate reductase (DHFR), correlated with their biological activity.

**Figure 4:**
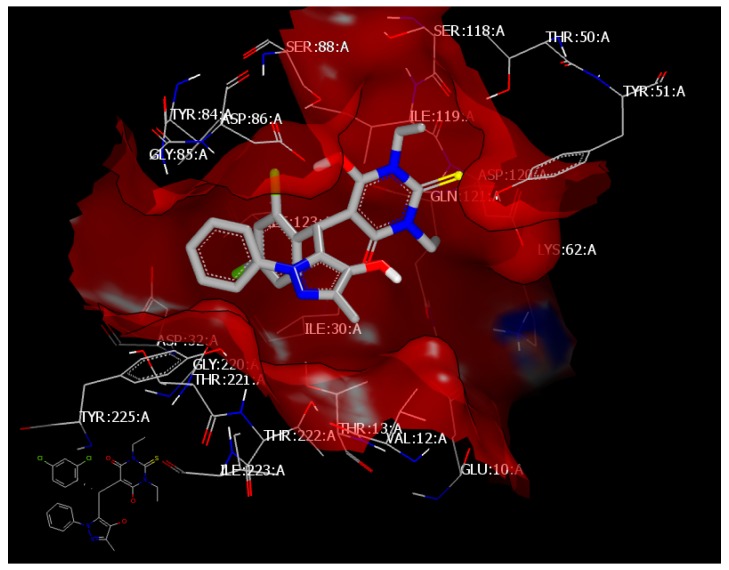
Visual representation of **4l** showed the hydrophobic–hydrophobic interaction with the binding site of 3Q70, as shown by Vida.

**Figure 5:**
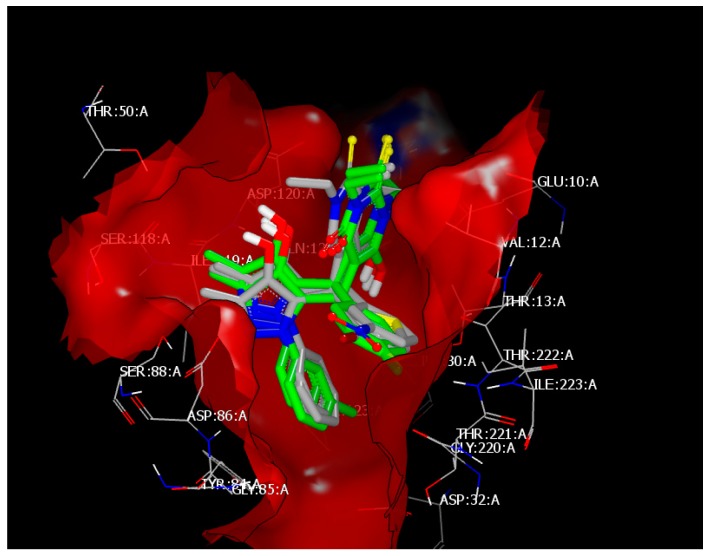
Visual representation of **4a** overlay with **4o**, **4d**, **4g**, and **4i**, showing the hydrophobic–hydrophobic interaction at the binding site of 3Q70, as shown by Vida.

Compound **4l** interacts with the receptor of dihydrofolate reductase (DHFR) (ID 3Q70) [36] through hydrophobic–hydrophobic interaction, with a consensus score of 24 (Figure 4). Similarly, compounds **4o** and **4a**, with consensus scores of 26 and 28, respectively, interact with the same receptor through hydrophobic–hydrophobic interaction and overlay with compounds **4c**, **4d**, **4g**, and **4i** (Figure 5).

#### 3.1.4. Molecular Docking as Gram-Positive Bacteria Antagonist

Molecular modeling gave us the comparative consensus scores of synthesized compounds with two targets: DNA topisomerase II (PDB ID 5BTC) [37] and gyrase B (PDB ID 4URM) proteins [38]. The consensus scores for the tested compounds with these two proteins are listed in Table 4.

#### 3.1.5. Docking with DNA Topisomerase II (5BTC)

**Figure 6:**
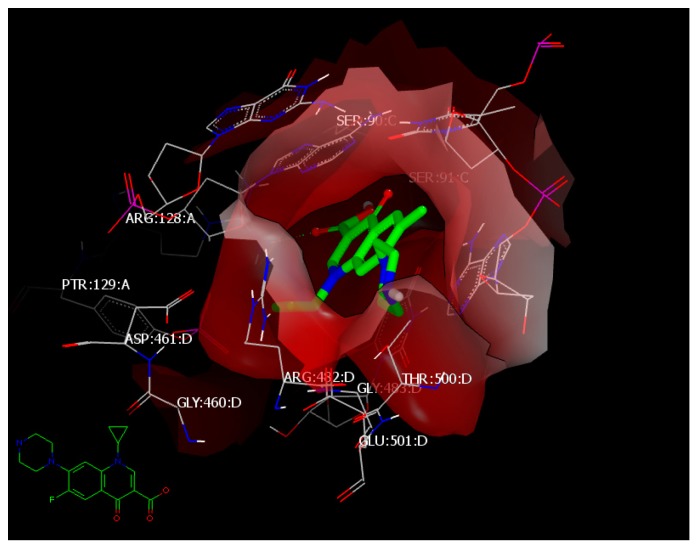
Visual representation of ciprofloxacin docked with 5BTC, showing hydrophobic–hydrophobic interaction and hydrogen bonding with ARG 128:A, as shown by Vida.

**Figure 7:**
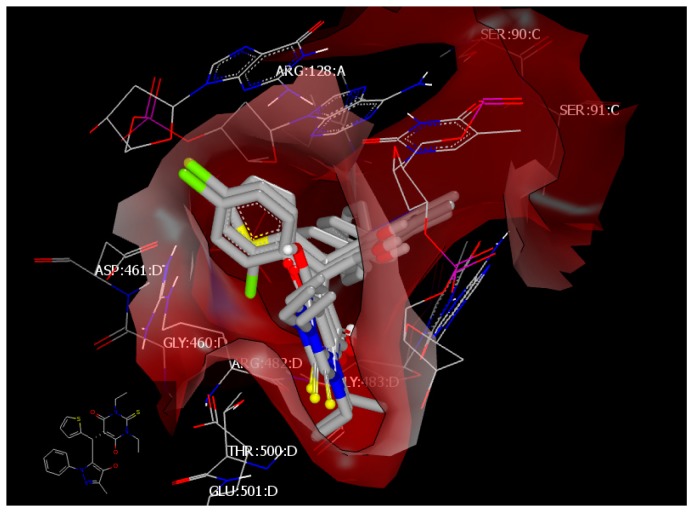
Visual representation of compounds **4c**, **4o**, and **4l** docked with 5BTC, showing no hydrogen bond interaction, as shown by Vida.

The standard drug ciprofloxacin has a consensus score of 1 through hydrophobic–hydrophobic interaction and forms HB with ARG:128:A through the oxygen of its carbonyl (Figure 6). The docking mode for the most active compounds showed hydrophobic–hydrophobic interaction with the receptor. Compound **4c** with a consensus score of 29, compound **4o** with a consensus score of 10, and compound **4l** with a consensus score of 54 showed exhibited hydrophobic–hydrophobic interactions and overlay each other (Figure 7). Our attention was directed to explore another kind of protein.

#### 3.1.6. With Gyrase B (PDB ID 4URM)

**Figure 8:**
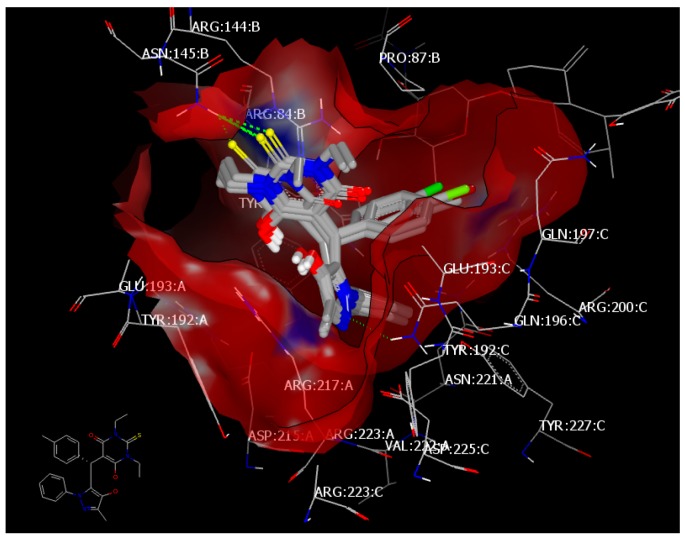
Visual representation of compound **4d** docked with 4URM and overlay with **4c**, **4a** and **4f**. The compounds showed hydrogen bonding between the sulfur of the pyrimidine ring and ASN 145:A, as shown by Vida.

**Figure 9:**
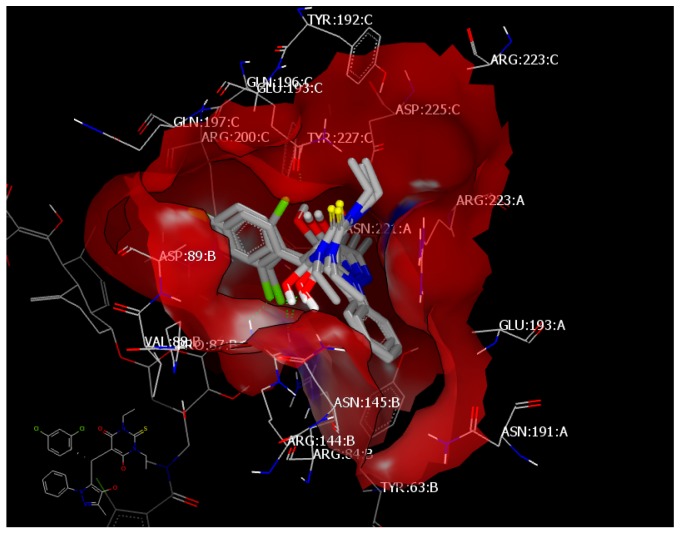
Visual representation of compounds **4l** and **4m** docked with 4URM without HB interaction and overlay with **4b**.

Ciprofloxacin forms a hydrogen bond with ASP 225:A through the NH of the piperidine moiety with a consensus score of 4. Compound **4c** (consensus score: 24) showed HB interaction with ASN 145:A through the sulfur atom of the thiobarbiturate ring and overlay with **4a**, **4d**, and **4f** with the same HB. However, **4a** showed extra HB with GLN 196:A through the N of pyrazole (Figure 8). Compounds **4l** (consensus score 17) and **4m** (consensus score 41) showed hydrophobic–hydrophobic interaction and overlay with **4b**; however, compound **4b** (consensus score: 24) exhibits three kinds of HB (Figure 9).

The antimicrobial activity of these compounds might be attributed to the presence of different pharmacophores in the molecules. The sulfur and OH groups in the substrates actively participate in the biological activity, as is corroborated by the formation of a hydrogen bond with the amino acid in the receptor. The site and kind of substituent located in the aldehyde part affects the dissociation rate, required for the liberation of free compounds from its salt form, and participates in the ligand–protein interaction. In this regard, the presence of an electron donating group (EDG) retards activity, while an electron withdrawing group (EWG) represents the best activity. Among the compounds having EWG, the para position is the best site for activity and the electronic and steric effect has a role in activity (compound **4f** is inactive). However, the difference in activity between compounds **4l** and **4m** could be explained by their 3D structures during the docking study by using Omega application. In compound **4m**, the chloride atom in position 6 was incorporated closely with the hydroxyl groups, while in the case of compound **4l** its presence at position para meant the chloride atom was not incorporated near the hydroxyl group. This evidence represents the importance of EWG at the para position (see the Supplementary Materials).

## 4. Materials and Methods

“All the chemicals were purchased from Sigma-Aldrich (Riedstraße, Germany) Fluka (Buchs, Switzerland), etc., and were used without further purification, unless otherwise stated. All melting points were measured on a Gallenkamp melting point apparatus (Bibby Scientific Limited, Beacon Road, Stone, Staffordshire, UK) in open glass capillaries and are uncorrected. IR Spectra were measured as KBr pellets on a Nicolet 6700 FT-IR spectrophotometer (Thermo Fisher Scientific, Madison, WI, USA). The NMR spectra were recorded on a Jeol-400 NMR spectrometer (Tokyo, Japan). ^1^H-NMR (400 MHz), and ^13^C-NMR (100 MHz) were run in deuterated dimethylsulphoxide (DMSO-*d*_6_). Chemical shifts (*δ*) are referred to in terms of ppm and J-coupling constants are given in Hz. Mass spectra were recorded on a Jeol of JMS-600 H (Santa Clara, CA, USA). Elemental analysis was carried out on an Elmer 2400 Elemental Analyzer (Waltham, MA, USA) in the CHN mode”.

### 4.1. General Procedure for Knoevenagel Condensation–Michael Addition for the Synthesis of ***4a–o*** (GP1)

A mixture of aldehyde **1** (1.5mmol), 1,3-diethyl-2-thioxodihydropyrimidine-4,6(1*H*,5*H*)-dione **2**, (1.5 mmol), 3-methyl-1-phenyl-1*H*-pyrazol-5(4*H*)-one (1.5mmol) and Et_2_NH (1.5 mmol, 155 μL) in 3 mL of degassed H_2_O was stirred at room temperature for 1–5 h until TLC showed complete disappearance of the reactants. The precipitate was removed by filtration and washed with ether (3 × 20 mL). The solid was dried to afford pure product **4a**–**o**.

*1,3-Diethyl-5-((4-fluorophenyl)(5-hydroxy-3-methyl-1-phenyl-1H-pyrazol-4-yl)methyl)-6-hydroxy-2-thioxo-2,3-dihydropyrimidin-4(1H)-one diethylaminium salt* **4a**: **4a** was prepared according to the general procedure (GP1) from *p*-flurobenzaldehyde, yielding a pink powder materilas. m.p.: 118 °C; IR (KBr, cm^−1^): 3445, 2989, 272, 2511, 1646, 1602, 1498, 1392, 1305, 1269; ^1^H-NMR (400 MHz, DMSO-*d*_6_): *δ* 17.65 (s, 1H, OH), 9.14 (bs, NH, NHEt_2_), 7.55–7.20 (m, 9H, Ph), 5.52 (s, 1H, benzyl-H), 4.57 (m, 4H, C*H*_2_CH_3_), 2.52 (q, 4H, *J* = 7.3 Hz, C*H*_2_CH_3_), 2.04 (s, 3H, CH_3_), 1.03 (t, 6H, *J* = 7.3 Hz, CH_2_C*H*_3_), 1.00 (t, 6H, *J* = 7.3 Hz, CH_2_C*H*_3_); ^13^C-NMR (100 MHz, DMSO-*d*_6_): *δ* = 193.2, 174.7, 164.0, 148.9, 146.5, 138.5, 129.0, 128.9, 128.2, 126.3, 122.2, 114.7, 114.5, 96.6, 43.4, 42.0, 15.2, 12.6, 12.5, 11.2; LC/MS (ESI): 280.1 [M]^+^ For C_18_H_17_FN_2_; Anal. for C_29_H_36_FN_5_O_3_S; calcd. C, 59.69; H, 6.01; F, 9.44; N, 11.60; S, 5.31; Found: C, 59.67; H, 6.01; F, 9.44; N, 11.63; S, 5.32.

*1,3-Diethyl-6-hydroxy-5-((5-hydroxy-3-methyl-1-phenyl-1H-pyrazol-4-yl)(phenyl)methyl)-2-thioxo-2,3-dihydropyrimidin-4(1H)-one diethylaminium salt* **4b**: **4b** was prepared according to the general procedure (GP1) from benzaldehyde, yielding faint orange materials. m.p.: 116 °C; IR (KBr, cm^−1^): 3448, 3023, 2982, 2785, 2508, 1580, 1502, 1410, 1389, 1264; ^1^H-NMR (400 MHz, DMSO-*d*_6_): *δ* 17.60 (s, 1H, OH), 7.55–7.13 (m, 10H, Ph), 5.53 (s, 1H, benzyl-H), 4.57 (m, 4H, C*H*_2_CH_3_), 2.32 (q, 4H, *J* = 7.3 Hz, C*H*_2_CH_3_), 2.10 (s, 3H, CH_3_), 1.21 (t, 6H, *J* = 7.3 Hz, CH_2_C*H*_3_), 0.90 (t, 6H, *J* = 7.3 Hz, CH_2_C*H*_3_); ^13^C-NMR (100 MHz, DMSO-*d*_6_): *δ* = 198.0, 174.8, 164.0, 163.6, 163.2, 151.4, 146.9, 138.0, 128.8, 127.9, 127.5, 126.9, 125.9, 121.9, 121.8, 91.2, 65.8, 42.1, 12.6, 12.2, 10.7; LC/MS (ESI): 262.1 [M]^+^ for C_18_H_18_N_2_; Anal. for C_29_H_37_N_5_O_3_S; calcd. C, 65.02; H, 6.96; N, 13.07; S, 5.99; Found: C, 65.03; H, 6.94; N, 13.05; S, 6.01.

*5-((4-Chlorophenyl)(5-hydroxy-3-methyl-1-phenyl-1H-pyrazol-4-yl)methyl)-1,3-diethyl-6-hydroxy-2-thioxo-2,3-dihydropyrimidin-4(1H)-one diethylaminium salt* **4c**: **4c** was prepared according to the general procedure (GP1) from *p*-cholorbenzaldehyde, yielding white materials. m.p.: 178 °C; IR (KBr, cm^−1^): 3453, 2981, 2873, 2495, 1686, 1582, 1487, 1438, 1386, 1267; ^1^H-NMR (400 MHz, DMSO-*d*_6_): *δ* 17.62 (s, 1H, OH), 9.33 (bs, NH, NHEt_2_), 7.24–6.96 (m, 10H, Ph), 5.80 (s, 1H, benzyl-H), 4.59 (m, 4H, C*H*_2_CH_3_), 3.32 (q, 4H, *J* = 7.3 Hz, C*H*_2_CH_3_), 3.29 (s, 3H, CH_3_), 3.03 (t, 6H, *J* = 7.3 Hz, CH_2_C*H*_3_), 1.29 (t, 6H, *J* = 7.3 Hz, CH_2_C*H*_3_); ^13^C-NMR (100 MHz, DMSO-*d*_6_): *δ* = 198.2, 174.8, 164.0, 163.6, 163.2, 151.4, 139.7, 139.1, 131.4, 128.3, 127.8, 96.8, 91.2, 44.1, 42.1, 34.2, 28.6, 12.4, 12.3, 11.3; LC/MS (ESI): 296.1 [M]^+^ for C_18_H_17_ClN_2;_ Anal. for C_29_H_36_ClN_5_O_3_S; calcd. C, 61.09; H, 6.36; Cl, 6.22; N, 12.28; S, 5.62; Found: C, 61.11; H, 6.34; Cl, 6.21; N, 12.31; S, 5.62.

*1,3-Diethyl-6-hydroxy-5-((5-hydroxy-3-methyl-1-phenyl-1H-pyrazol-4-yl)(p-tolyl)methyl)-2-thioxo-2,3-dihydropyrimidin-4(1H)-one diethylaminium salt* **4d**: **4d** was prepared according to the general procedure (GP1) from *p-*toulaldehyde, yielding orange materials (1.4 g, 2.97 mmol, 99%). m.p.: 193 °C; IR (KBr, cm^−1^): 3456, 3046, 2981, 2873, 2501, 1674, 1600, 1437, 1385, 1268; ^1^H-NMR (400 MHz, DMSO-*d*_6_): *δ* 17.63 (s, 1H, OH), 9.30 (bs, NH, NHEt_2_), 7.24–6.93 (m, 10H, Ph), 5.82 (s, 1H, benzyl-H), 4.59 (m, 4H, C*H*_2_CH_3_), 3.32 (q, 4H, *J* = 7.3 Hz, C*H*_2_CH_3_), 3.29 (s, 3H, CH_3_), 3.03 (t, 6H, *J* = 7.3 Hz, CH_2_C*H*_3_), 2.24 (s, 3H, CH_3_), 1.27 (t, 6H, *J* = 7.3 Hz, CH_2_C*H*_3_); ^13^C-NMR (100 MHz, DMSO-*d*_6_): *δ* = 196.2, 174.6, 164.0, 163.6, 163.1, 159.4, 151.4, 139.6, 139.3, 131.4, 128.8, 128.8, 126.2, 126.1, 97.1, 91.1, 44.3, 41.9, 34.2, 28.8, 20.9, 12.4, 12.2, 11.3; LC/MS (ESI): 276.1 [M]^+^ for C_19_H_20_N_2_; Anal. for C_30_H_39_N_5_O_3_S; calcd. C, 65.55; H, 7.15; N, 12.74; S, 5.83; Found: C, 65.54; H, 7.15; N, 12.73; S, 5.83.

*1,3-Diethyl-6-hydroxy-5-((5-hydroxy-3-methyl-1-phenyl-1H-pyrazol-4-yl)(m-tolyl)methyl)-2-thioxo-2,3-dihydropyrimidin-4(1H)-one diethylaminium salt* **4e**: **4e** was prepared according to the general procedure (GP1) from *m-*toulaldehyde, yielding white materials. m.p.: 200 °C; IR (KBr, cm^−1^): 3453, 3039, 2980, 2506, 1688, 1599, 1437, 1408, 1348, 1267; ^1^H-NMR (400 MHz, DMSO-*d*_6_): *δ* 17.59 (s, 1H, OH), 9.33 (bs, NH, NHEt_2_), 7.24–6.79 (m, 10H, Ph), 5.80 (s, 1H, benzyl-H), 4.62 (m, 4H, C*H*_2_CH_3_), 3.34 (q, 4H, *J* = 7.3 Hz, C*H*_2_CH_3_), 3.29 (s, 3H, CH_3_), 3.04 (t, 6H, *J* = 7.3 Hz, CH_2_C*H*_3_), 2.27 (s, 3H, CH_3_), 1.27 (t, 6H, *J* = 7.3 Hz, CH_2_C*H*_3_); ^13^C-NMR (100 MHz, DMSO-*d*_6_): *δ* = 196.2, 174.7, 163.7, 163.2, 152.3, 140.2, 137.5, 128.0, 126.9, 126.5, 123.4, 96.8, 91.5, 44.4, 44.1, 41.9, 34.6, 21.4, 12.4, 12.3, 11.3; LC/MS (ESI): 276.1 [M]^+^ for C_19_H_20_N_2_; Anal. for C_30_H_39_N_5_O_3_S; calcd. C, 65.55; H, 7.15; N, 12.74; S, 5.83; Found: C, 65.55; H, 7.15; N, 12.74; S, 5.85.

*5-((4-Bromophenyl)(5-hydroxy-3-methyl-1-phenyl-1H-pyrazol-4-yl)methyl)-1,3-diethyl-6-hydroxy-2-thioxo-2,3-dihydropyrimidin-4(1H)-one diethylaminium salt* **4f**: **4f** was prepared according to the general procedure (GP1) from *p*-bromobenzaldehyde, yielding beige materials. m.p.: 156 °C; IR (KBr, cm^−1^): 3431, 3006, 2981, 2509, 1603, 1579, 1501, 1484, 1411, 1390, 264; ^1^H-NMR (400 MHz, DMSO-*d*_6_): *δ* 17.61 (s, 1H, OH), 9.34 (bs, NH, NHEt_2_), 7.52–7.02 (m, 10H, Ph), 5.43 (s, 1H, benzyl-H), 4.54 (m, 4H, C*H*_2_CH_3_), 3.48 (q, 4H, *J* = 7.3 Hz, C*H*_2_CH_3_), 2.15 (q, 4H, *J* = 7.3 Hz, C*H*_2_CH_3_), 1.97 (s, 3H, CH_3_), 1.21 (t, 6H, *J* = 7.3 Hz, CH_2_C*H*_3_), 0.78 (t, 6H, *J* = 7.3 Hz, CH_2_C*H*_3_); ^13^C-NMR (100 MHz, DMSO-*d*_6_): *δ* = 146.7, 138.2, 131.1, 129.4, 128.8, 126.2, 121.9, 98.2, 65.8, 41.3, 15.2, 12.6, 12.2, 10.7; LC/MS (ESI): 340.1 [M]^+^ for C_18_H_17_BrN_2_; Anal. for C_29_H_36_BrN_5_O_3_S; calcd. C, 56.67; H, 5.90; Br, 13.00; N, 11.40; S, 5.22; Found: C, 56.67; H, 5.89; Br, 13.01; N, 11.43; S, 5.20.

*5-((3-Bromophenyl)(5-hydroxy-3-methyl-1-phenyl-1H-pyrazol-4-yl)methyl)-1,3-diethyl-6-hydroxy-2-thioxo-2,3-dihydropyrimidin-4(1H)-one diethylaminium salt* **4g**: **4g** was prepared according to the general procedure (GP1) from *m*-bromobenzaldehyde, yielding a pink powder materials. m.p.: 144 °C; IR (KBr, cm^−1^): 3432, 3060, 2981, 2743, 2505, 1611, 1591, 1501, 1404, 1264; ^1^H-NMR (400 MHz, DMSO-*d*_6_): *δ* 17.61 (s, 1H, OH), 9.34 (bs, NH, NHEt_2_), 7.52–7.02 (m, 10H, Ph), 5.43 (s, 1H, benzyl-H), 4.54 (m, 4H, C*H*_2_CH_3_), 3.48 (q, 4H, *J* = 7.3 Hz, C*H*_2_CH_3_), 2.15 (q, 4H, *J* = 7.3 Hz, C*H*_2_CH_3_), 1.97 (s, 3H, CH_3_), 1.21 (t, 6H, *J* = 7.3 Hz, CH_2_C*H*_3_), 0.78 (t, 6H, *J* = 7.3 Hz, CH_2_C*H*_3_); ^13^C-NMR (100 MHz, DMSO-*d*_6_): *δ* = 146.7, 138.2, 131.1, 129.4, 128.8, 126.2, 121.9, 98.2, 65.8, 41.3, 15.2, 12.6, 12.2, 10.7; LC/MS (ESI): 340.1 [M]^+^ for C_18_H_17_BrN_2_;Anal. for C_29_H_36_BrN_5_O_3_S; calcd. C, 56.67; H, 5.90; Br, 13.00; N, 11.40; S, 5.22; Found: C, 56.67; H, 5.89; Br, 13.01; N, 11.43; S, 5.20.

*1,3-Diethyl-6-hydroxy-5-((5-hydroxy-3-methyl-1-phenyl-1H-pyrazol-4-yl)(4-nitrophenyl)methyl)-2-thioxo-2,3-dihydropyrimidin-4(1H)-one diethylaminium salt* **4h**: **4h** was prepared according to the general procedure (GP1) from *p*-nitrobenzaldehyde, yielding yellow materials. m.p.: 108 °C; IR (KBr, cm^−1^): 3448, 2982, 2732, 2502, 1705, 1580, 1514, 1411, 1345, 1264; ^1^H-NMR (400 MHz, DMSO-*d*_6_): *δ* 17.61 (s, 1H, OH), 10.14 (bs, NH, NHEt_2_), 8.01 (dd, 2H, *J* = 7.3 Hz, 1.5Hz, Ph), 7.53 (dd, 2H, *J* = 7.3 Hz, 1.5 Hz, Ph) 7.48–7.2 (m, 5H, Ph), 5.55 (s, 1H, benzyl-H), 4.54 (m, 4H, C*H*_2_CH_3_), 2.37 (q, 4H, *J* = 7.3 Hz, C*H*_2_CH_3_), 2.05 (s, 3H, CH_3_), 1.27 (t, 6H, *J* = 7.3 Hz, CH_2_C*H*_3_), 0.92 (t, 6H, *J* = 7.3 Hz, CH_2_C*H*_3_); ^13^C-NMR (100 MHz, DMSO-*d*_6_): *δ* = 192.2, 174.7, 163.7, 163.2, 152.3, 146.6, 130.5, 129.0, 128.9, 128.4, 127.8, 126.3, 124.3, 123.2, 122.1, 103.3, 96.1, 43.4, 41.7, 34.7, 34.2, 12.5, 12.3, 10.7 ; LC/MS (ESI): 307.1 [M]^+^ for C_18_H_17_N_3_O_2_; Anal. for C_29_H_36_N_6_O_5_S; calcd. C, 59.98; H, 6.25; N, 14.47; S, 5.52; Found: C, 60.00; H, 6.26; N, 14.45; S, 5.53.

*1,3-Diethyl-6-hydroxy-5-((5-hydroxy-3-methyl-1-phenyl-1H-pyrazol-4-yl)(3-nitrophenyl)methyl)-2-thioxo-2,3-dihydropyrimidin-4(1H)-one* *diethylaminium salt* **4i**: **4i** was prepared according to the general procedure (GP1) from *m*-nitrobenzaldehyde, yielding yellow materials. m.p.: 136 °C; IR (KBr, cm^−1^): 3433, 2982, 2787, 2508, 1581, 1526, 1503, 1410, 1348, 1264; ^1^H-NMR (400 MHz, DMSO-*d*_6_): *δ* 17.61 (s, 1H, OH), 10.14 (bs, NH, NHEt_2_), 7.97 (s, 1H, Ph), 7.58–7.2 (m, 7H, Ph), 5.55 (s, 1H, benzyl-H), 4.60 (m, 4H, C*H*_2_CH_3_), 2.61 (q, 4H, *J* = 7.3 Hz, C*H*_2_CH_3_), 2.08 (s, 3H, CH_3_), 1.22 (t, 6H, *J* = 7.3 Hz, CH_2_C*H*_3_), 1.11 (t, 6H, *J* = 7.3 Hz, CH_2_C*H*_3_); ^13^C-NMR (100 MHz, DMSO-*d*_6_): *δ* = 192.2, 175.0, 163.7, 163.2, 152.3, 148.6, 130.5, 129.0, 128.9, 128.4, 127.8, 126.3, 124.3, 123.2, 122.1, 103.3, 96.1, 43.4, 42.2, 15.2, 12.6, 12.5, 11.1; LC/MS (ESI): 307.1 [M]^+^ for C_18_H_17_N_3_O_2_; Anal. for C_29_H_36_N_6_O_5_S; calcd. C, 59.98; H, 6.25; N, 14.47; S, 5.52; Found: C, 60.01; H, 6.26; N, 14.45; S, 5.54.

*1,3-Diethyl-6-hydroxy-5-((5-hydroxy-3-methyl-1-phenyl-1H-pyrazol-4-yl)(4-methoxyphenyl)methyl)-2-thioxo-2,3-dihydropyrimidin-4(1H)-one* *diethylaminium salt* **4j**: **4j** was prepared according to the general procedure (GP1) from anisaldehyde, yielding white crystalline materials. m.p: 112 °C; IR (KBr, cm^−1^): 3424, 2981, 2735, 2508, 1610, 1506, 1406, 1388, 1265; ^1^H-NMR (400 MHz, DMSO-*d*_6_): *δ* 17.71 (s, 1H, OH), 9.14 (bs, NH, NHEt_2_), 7.58–6.80 (m, 9H, Ph), 5.46 (s, 1H, benzyl-H), 4.60 (m, 4H, C*H*_2_CH_3_), 2.36 (q, 4H, *J* = 7.3 Hz, C*H*_2_CH_3_), 2.19 (s, 3H, CH_3_), 1.21 (t, 6H, *J* = 7.3 Hz, CH_2_C*H*_3_), 0.92 (t, 6H, *J* = 7.3 Hz, CH_2_C*H*_3_); ^13^C-NMR (100 MHz, DMSO-*d*_6_): *δ* = 194.2, 175.0, 163.7, 163.2, 152.3, 141.8, 138.5, 136.6, 128.8, 128.5, 127.8, 125.7, 121.8, 119.2, 114.2, 113.3. 113.2, 96.4, 91.5, 41.5, 34.5, 15.2, 12.6, 12.4, 11.0; LC/MS (ESI): 292.1 [M]^+^ for C_19_H_20_N_2_O; Anal. for C_30_H_39_N_5_O_4_S; calcd. C, 63.69; H, 6.95; N, 12.38; S, 5.67; Found: C, 63.70; H, 6.97; N, 12.41; S, 5.68.

*1,3-Diethyl-5-((4-trifluoromethylphenyl)(5-hydroxy-3-methyl-1-phenyl-1H-pyrazol-4-yl)methyl)-6-hydroxy-2-thioxo-2,3-dihydropyrimidin-4(1H)-one diethylaminium salt* **4k**: **4k** was prepared according to the general procedure (**GP1**) from *p*-trifluoromethyl benzaldehyde, yielding a pink powder materials. m.p.: 138 °C; IR (KBr, cm^−1^): 3441, 2982, 2787, 2505, 1615, 1579, 1420, 1390, 1265; ^1^H-NMR (400 MHz, DMSO-*d*_6_): *δ* 17.65 (s, 1H, OH), 9.14 (bs, NH, NHEt_2_), 7.55–7.24 (m, 9H, Ph), 5.52 (s, 1H, benzyl-H), 4.57 (m, 4H, C*H*_2_CH_3_), 2.52 (q, 4H, *J* = 7.3 Hz, C*H*_2_CH_3_), 2.04 (s, 3H, CH_3_),1.03 (t, 6H, *J* = 7.3 Hz, CH_2_C*H*_3_), 1.00 (t, 6H, *J* = 7.3 Hz, CH_2_C*H*_3_); ^13^C-NMR (100 MHz, DMSO-*d*_6_): *δ* = 193.2, 174.7, 164.0, 148.9, 146.5, 138.5, 129.0, 128.9, 128.2, 126.3, 122.2, 114.7, 114.5, 96.6, 43.4, 42.0, 15.2, 12.6, 12.5, 11.2; LC/MS (ESI): 330.13 [M]^+^ for C_19_H_17_F_3_N_2_; Anal. for C_30_H_36_F_3_N_5_O_3_S; calcd. C, 59.69; H, 6.01; F, 9.44; N, 11.60; S, 5.31; Found: C, 59.67; H, 6.01; F, 9.44; N, 11.63; S, 5.32.

*5-((2,4-Dichlorophenyl)(5-hydroxy-3-methyl-1-phenyl-1H-pyrazol-4-yl)methyl)-1,3-diethyl-6-hydroxy-2-thioxo-2,3-dihydropyrimidin-4(1H)-one diethylaminium salt* **4l**: **4l** was prepared according to the general procedure (GP1) from 2,4-dicholrobenzaldehyde, yielding an orange powder materials. m.p.: 132 °C; IR (KBr, cm^−1^): 3444, 2981, 2736, 2506, 1582, 1503, 1455, 1410, 1388, 1264; ^1^H-NMR (400 MHz, DMSO-*d*_6_): *δ* 17.65 (s, 1H, OH), 7.55–7.21 (m, 9H, Ph), 5.41 (s, 1H, benzyl-H), 4.49 (m, 4H, C*H*_2_CH_3_), 2.53 (q, 4H, *J* = 7.3 Hz, C*H*_2_CH_3_), 2.17 (s, 3H, CH_3_), 1.80 (bs, NH, NHEt_2_), 1.20 (t, 6H, *J* = 7.3 Hz, CH_2_C*H*_3_), 1.00 (t, 6H, *J* = 7.3 Hz, CH_2_C*H*_3_); ^13^C-NMR (100 MHz, DMSO-*d*_6_): *δ* = 195.2, 174.8, 164.0, 148.7, 146.5, 138.5, 129.0, 128.9, 128.2, 126.3, 122.2, 114.7, 114.5, 96.6, 43.2, 41.5, 15.4, 12.9, 12.7, 11.2; LC/MS (ESI): 330.07 [M]^+^ for C_18_H_16_Cl_2_N_2_; Anal. for C_29_H_35_Cl_2_N_5_O_3_S; calcd. C, 57.61; H, 5.84; Cl, 11.73; N, 11.58; S, 5.30; Found: C, 57.60; H, 5.85; Cl, 11.70; N, 12.01; S, 5.31.

*5-((2,4-Dichlorophenyl)(5-hydroxy-3-methyl-1-phenyl-1H-pyrazol-4-yl)methyl)-1,3-diethyl-6-hydroxy-2-thioxo-2,3-dihydropyrimidin-4(1H)-one diethylaminium salt* **4m**: **4m** was prepared according to the general procedure (GP1) from 2,6-dicholrobenzaldehyde, yielding a pink powder materials. m.p.: 171 °C; IR (KBr, cm^−1^): 3448, 2932, 2980, 2755, 2506, 1582, 1499, 1407, 1371, 1263; ^1^H-NMR (400 MHz, DMSO-*d*_6_): *δ* 17.30 (s, 1H, OH), 11.9 (bs, NH, NHEt_2_), 7.74 (d, 2H, *J* = 7.3 Hz, Ph), 7.37 (t, 1H, *J* = 7.3 Hz, Ph), 7.24–7.01 (m, 9H, Ph), 5.74 (s, 1H, benzyl-H), 4.37 (m, 4H, C*H*_2_CH_3_), 2.90 (q, 4H, *J* = 7.3 Hz, C*H*_2_CH_3_), 1.96 (s, 3H, CH_3_), 1.11 (t, 12H, *J* = 7.3 Hz, CH_2_C*H*_3_); ^13^C-NMR (100 MHz, DMSO-*d*_6_): *δ* = 193.8, 173.9, 161.7, 153.1, 146.6, 138.5, 135.7, 129.5, 128.7, 127.0, 124.4, 120.0, 94.7, 43.1, 42.0, 31.8, 12.5, 12.4, 11.0; LC/MS (ESI): 330.07 [M]^+^ for C_18_H_16_Cl_2_N_2_; Anal. for C_29_H_35_Cl_2_N_5_O_3_S; calcd. C, 57.61; H, 5.84; Cl, 11.73; N, 11.58; S, 5.30; Found: C, 57.60; H, 5.85; Cl, 11.70; N, 12.01; S, 5.31.

*1,3-Diethyl-6-hydroxy-5-((5-hydroxy-3-methyl-1-phenyl-1H-pyrazol-4-yl)(naphthalen-2-yl)methyl)-2-thioxo-2,3-dihydropyrimidin-4(1H)-one diethylaminium salt* **4n**: **4n** was prepared according to the general procedure (GP1) from naphthaldehyde, yielding white crystalline materials. m.p.: 118 °C; IR (KBr, cm^−1^): 3443, 3052, 2981, 2872, 2737, 2507, 1582, 1503, 1454, 1410, 1388, 1264; ^1^H-NMR (400 MHz, DMSO-*d*_6_): *δ* 17.65 (s, 1H, OH), 9.30 (bs, NH, NHEt_2_), 7.75–7.05 (m, 12H, Ph), 5.68 (s, 1H, benzyl-H), 4.61 (m, 4H, C*H*_2_CH_3_), 2.40 (q, 4H, *J* = 7.3 Hz, C*H*_2_CH_3_), 2.13 (s, 3H, CH_3_), 1.21 (t, 6H, *J* = 7.3 Hz, CH_2_C*H*_3_), 0.87 (t, 6H, *J* = 7.3 Hz, CH_2_C*H*_3_); ^13^C-NMR (100 MHz, DMSO-*d*_6_): *δ* = 193.8, 173.9, 161.7, 153.1, 146.6, 138.5, 135.7, 129.5, 128.7, 127.0, 124.4, 120.0, 94.7, 43.1, 42.0, 31.8, 12.5, 12.4, 11.0; LC/MS (ESI): 312 [M]^+^ for C_22_H_20_N_2_; Anal. for C_33_H_39_N_5_O_3_S; calcd C, 67.66; H, 6.71; N, 11.96; S, 5.47; Found: C, 67.67; H, 6.70; N, 11.95; S, 5.48.

*1,3-Diethyl-6-hydroxy-5-((5-hydroxy-3-methyl-1-phenyl-1H-pyrazol-4-yl)(thiophen-2-yl)methyl)-2-thioxo-2,3-dihydropyrimidin-4(1H)-one diethylaminium salt* **4o**: **4o** was prepared according to the general procedure (GP1) from thiophenldehyde, yielding a brown powder materials. m.p.: 122 °C; IR (KBr, cm^−1^): 3437, 2932, 2980, 2736, 2509, 1598, 1502, 1410, 1389, 1329, 1264; ^1^H-NMR (400 MHz, DMSO-*d*_6_): *δ* 17.30 (s, 1H, OH), 8.38 (bs, NH, NHEt_2_), 7.87 (d, 2H, *J* = 7.3 Hz, thiophene), 7.70 (d, 1H, *J* = 7.3 Hz, thiophene), 7.45–7.07 (m, 6H, Ph), 5.55 (s, 1H, benzyl-H), 4.44 (m, 4H, C*H*_2_CH_3_), 2.89 (q, 4H, *J* = 7.3 Hz, C*H*_2_CH_3_), 2.28 (s, 3H, CH_3_), 2.19 (t, 6H, *J* = 7.3 Hz, CH_2_C*H*_3_), 1.12 (t, 6H, *J* = 7.3 Hz, CH_2_C*H*_3_); ^13^C-NMR (100 MHz, DMSO-*d*_6_): *δ* = 193.8, 173.9, 163.2, 161.7, 152.2, 148.1, 148.0, 147.5, 137.3, 136.3, 135.7, 128.9, 128.6, 128.4, 128.2, 119.4, 101.9, 101.8, 97.3, 79.8, 43.2, 41.2, 34.5, 21.0, 12.8, 12.6, 11.012.4, 10.8; LC/MS (ESI): 268.1 [M]^+^ for : C_16_H_16_N_2_S; Anal. for C_27_H_35_N_5_O_3_S_2_; calcd. C, 59.86; H, 6.51; N, 12.93; S, 11.84; Found: C, 59.86; H, 6.51; N, 12.90; S, 11.83.

### 4.2. Agar Cup Plate Method

“The tested bacterial strains were grown in Cation Adjustment Mueller–Hinton (CAMH) broth (Merck^®^, Darmstadt, Germany), while *C. albicans* strain was grown in Sabauraud Dextrose Broth (SDB) to mid-log phase. The bacterial and fungal suspension was measured by spectrophotometery using Spectrophotometer (LKB^®^ Ultrospec, Madison, WI, USA) t 625 nm to give absorbance 0.12 (1 × 10^8^ CFU/mL). The suspension was diluted 1:100 in CAMH broth to obtain 1 × 10^6^ CFU/mL. This suspension was swabbed on a CAMH agar plate (Merck^®^, Darmstadt, Germany) and allowed to dry completely. Mueller–Hinton Agar and Sabauraud Dextrose Agar were used for bacteria and fungi, respectively. Four wells (7 mm in diameter) were made in agar plate using a cork borer. One milliliter of stock solution (5120 μg/mL) was diluted two-fold in 1 mL DMSO to obtain 2560 μg/mL. One hundred microliters (256 µg) of the tested compound was poured into the well using a calibrated pipette. The plates were kept in a refrigerator at 4 °C for half an hour to allow diffusion of the compound in the agar. Then, the plates were incubated at 37 °C for 24 h. After the incubation period, the diameter of the inhibition zone was measured and recorded in mm with the aid of a ruler. Ciprofloxacin (10 µg/cup) and fluconazole (10 µg/mL) were used as positive controls for antibacterial and antifungal activity, respectively. The experiment was carried out in duplicate and the mean diameter was taken” [39].

### 4.3. Determination of MIC

“MIC was determined for the compounds that showed antimicrobial activity by cup plate method. Briefly, 2 mL of CAMH broth (for bacterial strains) and 2 mL of SAB (for fungal strain) were dispensed into 7-mL Peju sterile tubes. For each compound, 14 tubes were used. Tubes 13 and 14 were used as a positive growth control (no tested compound) and a negative control for medium sterility (no microorganisms), respectively. One milliliter of stock solution (5120 μg/mL) was 10-fold diluted in 9 mL CAMH to obtain 512 μg/mL. Two milliliters of the tested compounds (512 μg/mL) were pipetted into the first tube and mixed well. Then 2 mL were withdrawn from the 1st tube and added to the 2nd tube to make a two-fold dilution. This procedure was repeated down to the 12th tube to reach a concentration of 0.125 μg/mL. Two milliliters were discarded from the 12th tube. A volume of 2 mL of inoculums (1 × 10^6^ CFU/mL) was added to all tubes except number 14 to give a final concentration of 1 × 10^6^ CFU/mL. Ciprofloxacin and fluconazole were used as positive controls for antibacterial and antifungal activity, respectively. The inoculated tubes were incubated at 37 °C for 20 h. After the incubation period, the results of MIC were recorded manually and interpreted according to the guidelines of the British Society of Antimicrobial Chemotherapy (BSAC)” [39].

### 4.4. Docking Study

The docking study was performed using the OpenEye program [40] and its applications Omega, Oedcocking, Fred, and Vida. This software package is able to perform consensus scoring, which is an essential filtering technique used to obtain more accurate predictions, i.e., the lower the consensus score, the better the binding affinity of the ligands towards the receptor. Both the ligand input file (compile all .pdb files) and the receptor input file were passed into Fred to perform the molecular docking simulations. Multiple scoring functions were employed in order to obtain a consensus structure and score in the final output. A virtual library of target compounds and fluconazole as the standard drug for antifungal activity and ciprofloxacin as the standard for Gram-positive antibacterial activity was prepared using Chem Office 2012 (OpenEye Scientific software Inc. (Santa Fe, NM, USA) and energy minimized with PM3 minimization. All calculations were performed on a PC running Windows 7 ultimate. Two different target proteins were downloaded from the Protein Data Bank, namely Lanosterol 14 α-demethylase (CYP51A1) (PDB ID 4WMZ) and secreted aspartic protease (PDB ID 3Q70), and were chosen as antifungal targets against *Candia albicans*. In order to understand the antibacterial activities of these newly synthesized compounds, DNA topisomerase II (PDB ID 5BTC) and gyrase B (PDB ID 4URM) were selected as antibacterial targets. The catalytic domain for each protein was prepared for docking using Open Eye (OpenEye Scientific software Inc. (Santa Fe, NM, USA).

#### 4.4.1. Generation of Conformers Using OMEGA

OMEGA (OpenEye Scientific software Inc., Santa Fe, NM, USA) was used to generate multi-conformer structure databases with high speed and reliability, in order to achieve flexible ligand docking, to be used in the docking simulations. The energy-minimized structures were then converted into .pdb files, maintaining all heavy atoms. Each .pdb file was concatenated into one continuous .pdb file to be used as an input for Omega.

#### 4.4.2. Receptor (PDB file) Preparation Using OEDCKING Application

The receptor PDB files were downloaded from the Protein Data Bank (PDB): Lanosterol 14 α-demethylase (CYP51A1) (PDB ID 4WMZ), and secreted aspartic protease (PDB ID 3Q70), and were chosen as antifungal targets against *Candia albicans*. Two different target proteins were downloaded from the Protein Data Bank in order to understand the antibacterial activities of these newly synthesized compounds. DNA topisomerase II (PDB ID 5BTC), and gyrase B (PDB ID 4URM) were selected as antibacterial targets and were prepared using the make_receptor command. This step will be followed by finding the potential binding sites on the receptor of interest. The application uses a molecular probe to comb the receptor molecule, identifying all the possible binding interactions. The application allows you to create a grid box in the mode selection pane and adjust its size using the mode controls. The box size should never exceed 50,000–60,000 A°.

#### 4.4.3. FRED Docking Application

Both the ligand input file and the receptor input file were passed into FRED to perform the molecular docking simulations. Multiple scoring functions were employed in order to obtain a consensus structure and score in the final output.

#### 4.4.4. Vida Application

Vida is a graphical user interface that visualizes, analyzes, and manages corporate collections of molecular structures and information. Snapshots were taken to visualize and obtain the main interaction forces between the analogs and the receptor of interest.

## 5. Conclusions

A series of pyrazole-thiobarbituric acid derivatives (**4a**–**o**) were synthesized using a one-pot method with a broad substrate scope under mild reaction conditions in water, mediated by NHEt_2_. The compounds were evaluated for their antifungal and antibacterial activity. The chemical structures of the newly synthesized molecules were characterized by spectroscopic methods (IR, ^1^H-NMR, ^13^C-NMR, MS) and elemental analysis. The results of the current study revealed that compounds **4h** and **4l** were the most active against *C. albicans*, with an MIC value of 4 µg/L. Next were compounds **4a** and **4o**, which showed activity of MIC 8 µg/L, as compared with the reference drug Fluconazole with a MIC value of 0.5 µg/L. Furthermore, all synthesized compounds were docked inside the active site for two kinds of proteins, namely Lanosterol 14 α-demethylase (CYP51A1) (PDB ID 4WMZ) and secreted aspartic protease (PDB ID 3Q70). Compound **4h** docked with 4WMZ and showed hydrogen bonding interactions with THR 318:A, a binding interaction similar to that of standard Fluconazole. Most of the compounds showed hydrophobic–hydrophobic interaction towards the binding site of 3Q70 and overlay each other.

However, among the synthesized compounds, compound **4c** exhibited marked activity against *S. aureus* and *E. faecalis*, with MIC values of 16 µg/L. Compounds **4l** and **4o** were the most active compounds against *B. subtilis* with MIC = 16 µg/L in comparison to standard ciprofloxacin, with MIC values of <0.25 µg/L against all tested bacteria. The target compounds were subjected to dock with DNA topisomerase II (PDB ID 5BTC) and gyrase B (PDB ID 4URM) proteins to explore their mode of interaction in Grampositive bacteria.

Based on a docking study of the antimicrobial activity of the tested compounds, the sulfur atom and OH functionality in the thiobarbiturate moiety, the OH group in the pyrazole fragment, and the presence of an EWG group in the *p*-position of the aryl part participate in the compound’s interaction with the tested receptors. These pharmacophores may contribute, at least in part, to the activity of the tested compounds as antimicrobial agents.

## Figures and Tables

**Table 1:** Synthesis of **4a**–**o** with different various aldehydes ^a^.

#	4	R	Yield (%) ^b^
1	**4a**	*p*-FPh	76
2	**4b**	Ph	83
3	**4c**	*p*-ClPh	84
4	**4d**	*p*-CH_3_Ph	73
5	**4e**	*m*-CH_3_Ph	78
6	**4f**	*p*-BrPh	88
7	**4g**	*m*-BrPh	73
8	**4h**	*p*-NO_2_Ph	73
9	**4i**	*m*-NO_2_Ph	72
10	**4j**	*p*-CH_3_OPh	69
11	**4k**	*p*-CF_3_Ph	63
12	**4l**	2,4-Cl_2_Ph	68
13	**4m**	2,6-Cl_2_Ph	65
14	**4n**	2-Naphthaldehyde	67
15	**4o**	Thiophene	78

^a^ All reactions were carried out with aldehyde **1** (1.5 mmol), 1,3-diethyl-2-thioxodihydropyrimidine-4,6(1*H*,5*H*)-dione **2**, (1.5 mmol), 3-methyl-1-phenyl-1*H*-pyrazol-5(4*H*)-one (1.5 mmol) and amine (1.5 mmol) in water (1.5 mL) for the specified time. ^b^ Yield of isolated product.

**Table 2:** Results of cup-plate method and minimal inhibitory concentrations of the compounds that show antimicrobial activity.

#	Compounds	Gram-Positive Bacteria						Yeast	
		*S. aureus* ATCC 29213		*E. faecalis* ATCC 29212		*B. subtilis* ATCC 10400		*C. albicans* ATCC 2091	
		CPM (mm)	MIC (µg/L)	CPM (mm)	MIC (µg/L)	CPM (mm)	MIC (µg/L)	CPM (mm)	MIC (µg/L)
1	**4a**	13	32	15	32	12	32	18	8
2	**4b**	12	32	12	32	10	32	16	16
3	**4c**	14	16	12	16	11	32	16	16
4	**4d**	Nil	128	9	128	11	64	11	64
5	**4e**	Nil	128	10	64	11	64	12	64
6	**4f**	Nil	128	10	64	11	64	15	32
7	**4g**	13	32	12	64	11	64	14	32
8	**4h**	13	32	16	32	11	64	20	4
9	**4i**	14	32	16	32	13	32	14	32
10	**4j**	14	32	18	16	13	32	17	16
11	**4k**	13	64	10	32	11	32	16	16
12	**4l**	13	32	20	32	15	16	21	4
13	**4m**	13	32	24	32	16	32	15	16
14	**4n**	14	32	11	32	11	32	16	16
15	**4o**	14	32	Nil	32	11	16	17	8
Standard	Ciprofloxacin	27	≤0.25	24	≤0.25	25	≤0.25	ND	ND
	Fluconazole	ND	ND	ND	ND	ND	ND	28	0.5

**Table 3:** Molecular modeling consensus score for the tested compounds and Fluconzole.

No.	R	Consensus Score	
		4WMZ	3Q70
Fluconazole	-	57	17
**4l**	2,4-Cl_2_Ph	48	24
**4h**	*p*-NO_2_Ph	55	25
**4o**	Thiophene	21	42
**4n**	2-Naphthalde	23	21
**4i**	*m*-NO_2_Ph	35	28
**4a**	*p*-FPh	14	28
**4g**	*m*-BrPh	15	31
**4d**	*p*-CH_3_Ph	36	35
**4b**	Ph	20	37
**4e**	*m*-CH_3_Ph	11	37
**4f**	*p*-BrPh	22	38
**4m**	2,6-Cl_2_Ph	28	41
**4c**	*p*-ClPh	33	45
**4k**	*p*-CF_3_Ph	34	47
**4j**	*p*-CH_3_OPh	40	5

**Table 4:** Molecular modeling consensus score for the tested compounds and Ciprofloxacin.

No.	R	Consensus Score	
		5BTC	4URM
Ciprofloxacin	-	1	4
**4o**	Thiophene	10	24
**4b**	Ph	17	24
**4j**	*p*-CH_3_OPh	18	30
**4e**	*m*-CH_3_Ph	19	33
**4d**	*p*-CH_3_Ph	23	22
**4h**	*p*-NO_2_Ph	23	55
**4c**	*p*-ClPh	29	24
**4f**	*p*-BrPh	29	29
**4a**	*p*-FPh	36	31
**4g**	*m*-BrPh	46	29
**4i**	*m*-NO_2_Ph	47	37
**4k**	*p*-CF_3_Ph	48	59
**4l**	2,4-Cl_2_Ph	54	17
**4n**	2-Naphthalde	54	37
**4m**	2,6-Cl_2_Ph	60	41

## Supplementary Materials

Supplementary materials can be accessed at: http://www.mdpi.com/1420-3049/21/10/1337/s1.
