# Supplementary material for: Synthesis of Pyrazole-Thiobarbituric Acid Derivatives: Antimicrobial Activity and Docking Studies
Source: Molecules. 2016 Oct 9;21(10):1337. doi: 10.3390/molecules21101337 (PMC6274314; doi:10.3390/molecules21101337)
Supplement: Supplementary file 1 [file molecules-21-01337-s001.pdf]

Supplementary Materials: Synthesis of Pyrazole-Thiobarbituric Acid Derivatives: Antimicrobial Activity and Docking Studies

Yaseen A. M. M. Elshaier, Assem Barakat, Bander M. Al-Qahtany, Abdullah Mohammed Al-Majid and Mohamed H. Al-Agamy

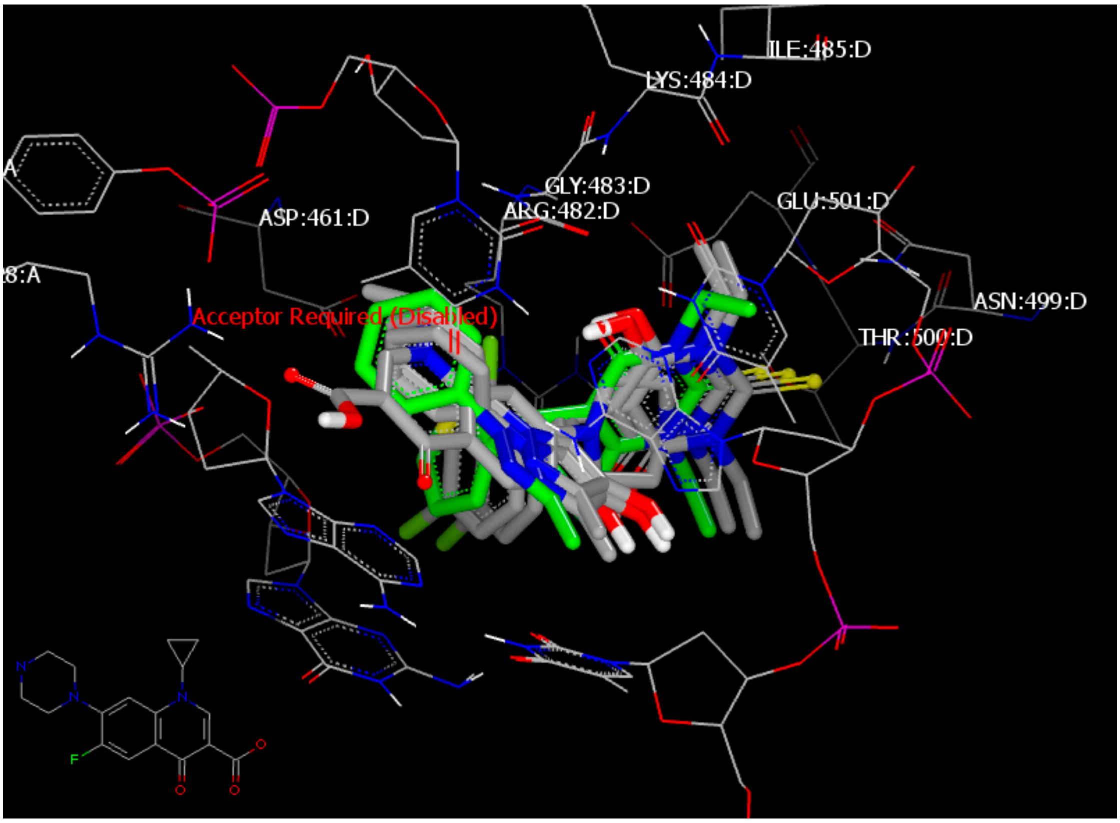

Figure S1. Ciprofloxacin and compounds **4c**, **4l**, and **4o** docked and overlay each other in the PD ID 5BTC receptor.

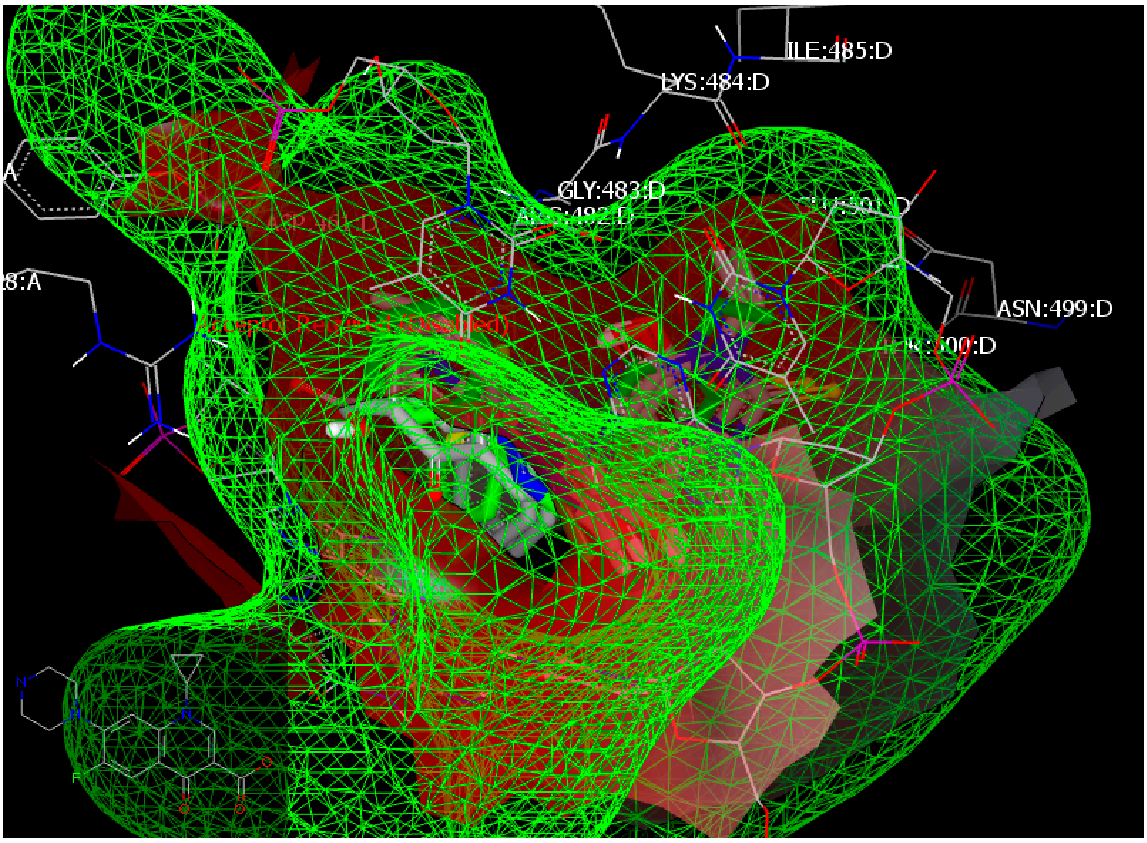

Figure S2. Ciprofloxacin and compounds **4c**, **4l**, and **4o** docked inside the grid for active site in the PD ID 5BTC receptor.

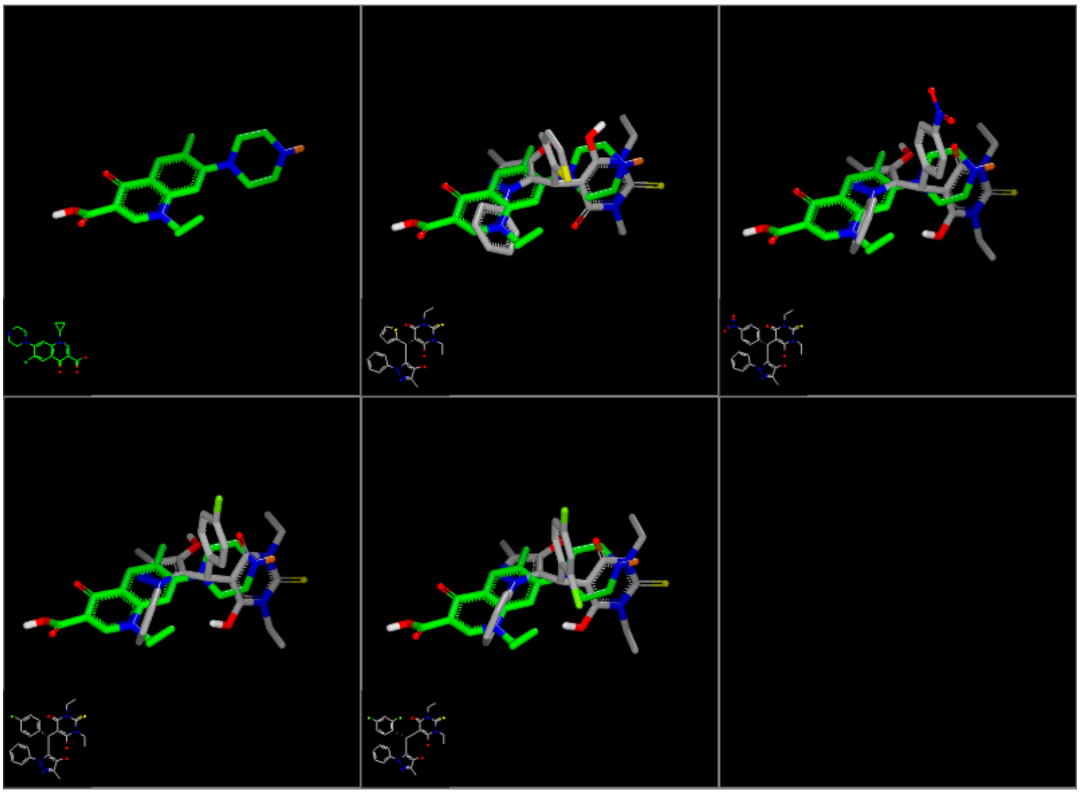

Figure S3. Shape comparison for compounds **4l**, **4o**, **4c**, and **4h** with ciprofloxacin, in 5BTC using ROCS application.

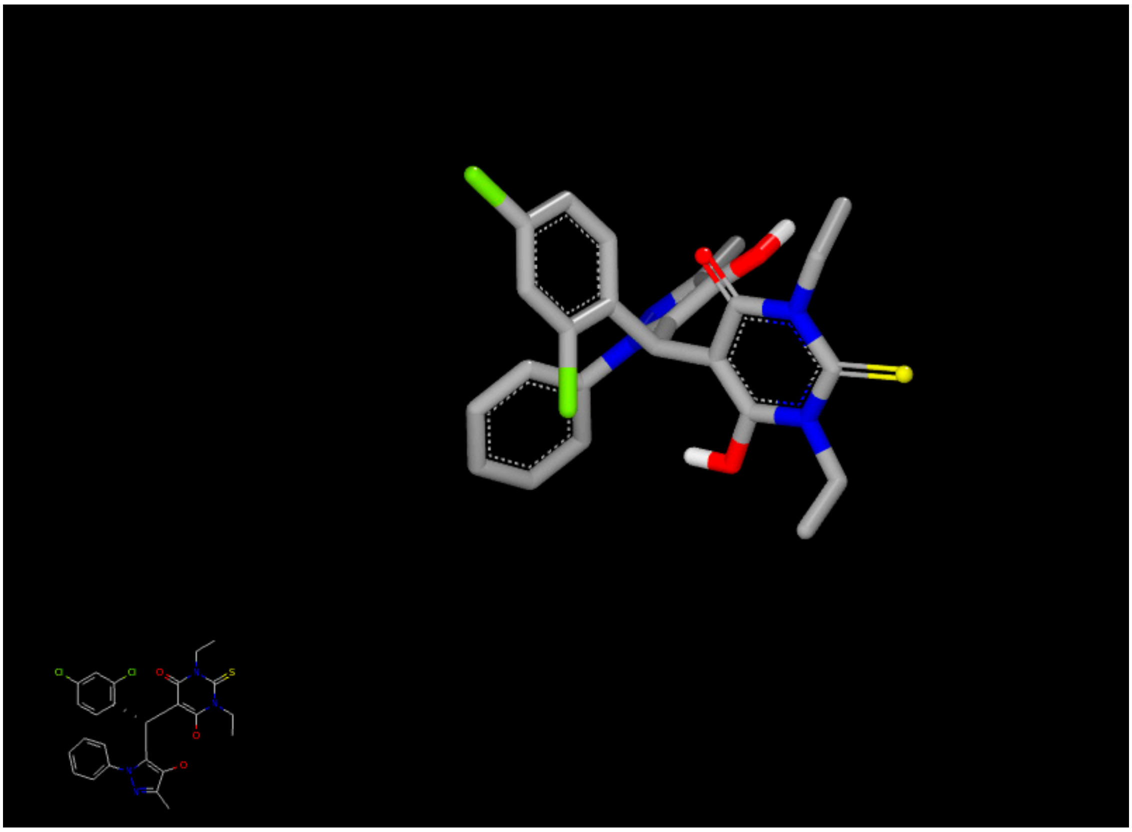

Figure S4. 3D structure for compound **4l** generated by Omega.

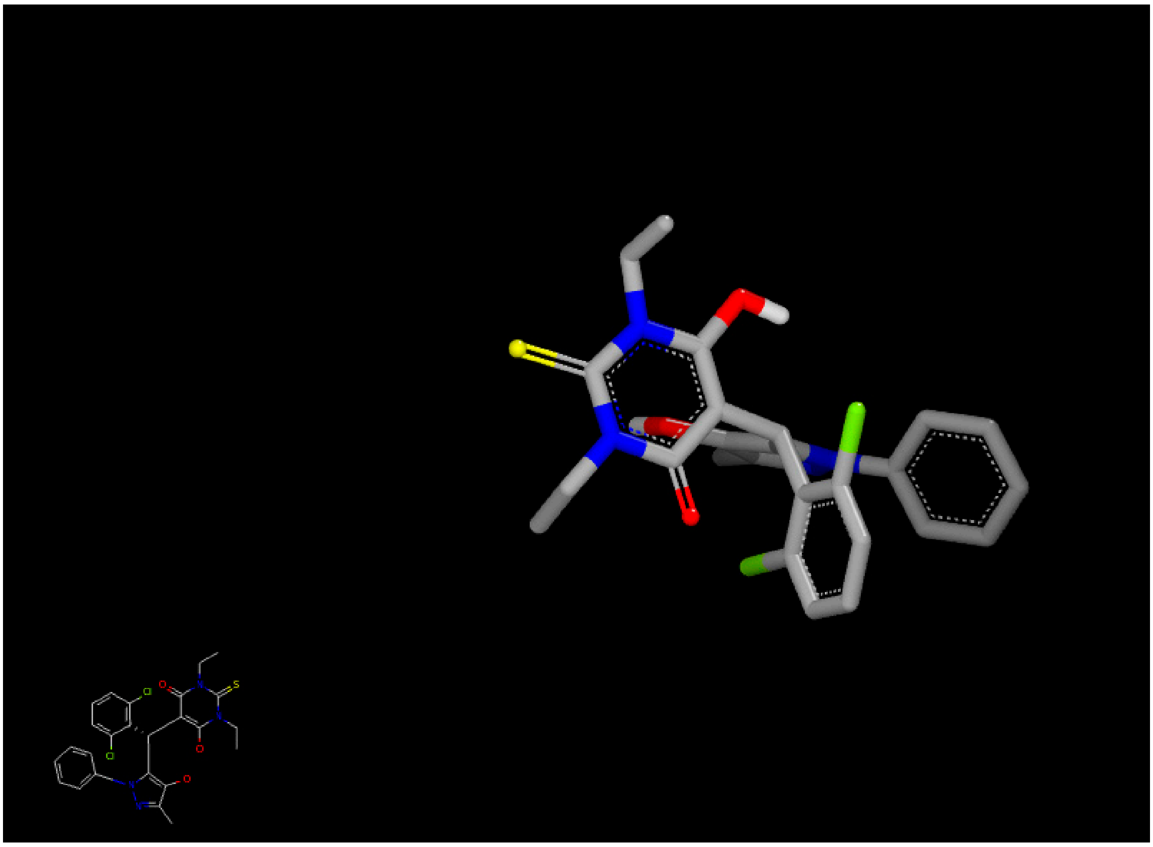

Figure S5. 3D structure for compound **4m** generated by Omega.
